# Lipoprotein FtsB in *Streptococcus pyogenes* Binds Ferrichrome in Two Steps with Residues Tyr137 and Trp204 as Critical Ligands

**DOI:** 10.1371/journal.pone.0065682

**Published:** 2013-06-20

**Authors:** Hui Li, Nan Li, Qian Xu, Chuanle Xiao, Hongcui Wang, Zhong Guo, Jing Zhang, Xuesong Sun, Qing-Yu He

**Affiliations:** Key Laboratory of Functional Protein Research of Guangdong Higher Education Institutes, Institute of Life and Health Engineering, College of Life Science and Technology, Jinan University, Guangzhou, China; The Chinese University of Hong Kong, China

## Abstract

Lipoprotein FtsB is a component of the FtsABCD transporter that is responsible for ferrichrome binding and uptake in the Gram-positive pathogen *Streptococcus pyogenes*. In the present study, FtsB was cloned and purified from the bacteria and its Fch binding characteristics were investigated in detail by using various biophysical and biochemical methods. Based on the crystal structures of homogeneous proteins, FtsB was simulated to have bi-lobal structure forming a deep cleft with four residues in the cleft as potential ligands for Fch binding. With the assistance of site-directed mutagenesis, residue Trp204 was confirmed as a key ligand and Tyr137 was identified to be another essential residue for Fch binding. Kinetics experiments demonstrated that Fch binding in FtsB occurred in two steps, corresponding to the bindings to Tyr137 at N-lobe and Trp204 from C-lobe, respectively, and so that closing the protein conformation. Without either residue Tyr137 or Trp204, Fch binding in the protein as mutants Fch-Y137A and Fch-W204A may have a loose conformation, resembling the apo-proteins in proteolysis resistance and migration behaviors in native gel. This study revealed the inconsistence in the key amino acids among Fch-binding proteins from Gram-positive and –negative bacteria, providing interesting findings for understanding the differences between Gram-positive and –negative bacteria in the mechanism of iron uptake via siderophore (Fch) binding and transport.

## Introduction

Iron is essential for the survival of almost all bacteria [1]. In the neutral and aerobic environment, free iron is quite limited (∼10^−18^ M) and is further sequestrated by carrier and storage proteins in host body [2]. To overcome this problem, some bacteria produce cell surface receptors of transferrin and lactoferrin to extract iron from these iron transporters, while others secrete siderophores (typically <1 kDa) to scavenge iron from the host environments [3]. Siderophores possess either phenolate or carboxylate oxygens that can bind Fe^3+^ with a high affinity (>10^30^ M^−1^). Siderophores are classified into catecholates, hydroxamates and hydroxycarboxylate based on their structures [4]. Ferrichrome (Fch) belongs to the hydroxamate type and is synthesized by some fungus such as *Ustilago sphaerogena*. Iron-free Fch binds Fe^3+^ in an octahedral conformation with three deprotonated hydroxyl groups and three carbonyl oxygens of the hydroxamic acid moieties. Transport of siderophores across the cell membrane is mediated by an ATP binding cassette (ABC) system [5], [6], [7].

*Streptococcus pyogenes* (*S. pyogenes*), one of the Group A *Streptococcus* (GAS), is an important Gram-positive human pathogen, causing a variety of diseases including pharyngitis, scarlet fever, skin diseases and invasive deep-tissue infections [8]. *S. pyogenes* utilizes hemoglobin, transport proteins and a multi-metal transport system to acquire iron [9]. *S. pyogenes* genome encodes three ABC transporters, *i.e*. HtsABC (or named as SiaABC), MtsABC and FtsABCD, which are supposed to transport heme, Fe^3+^ and Fch, respectively. *S. pyogenes* can use Fch as an iron source through the FtsABCD transporter that contains ATP-binding protein FtsA, lipoprotein FtsB and permease FtsC/D. The lipid moiety of FtsB is attached to the cell membrane of *S. pyogenes*. Fch is firstly captured by the lipoprotein FtsB with 1∶1 stoichiometry, and then transferred to the permease FtsC/D to go across the cell membrane using energy derived from the hydrolysis of ATP by FtsA. The expression level of FtsB increases when both *htsA* and *mtsA* genes were knocked out in an iron deficient environment [10], [11]. Apart from these observations, little is known about the biochemical and physiological details of FtsB including its Fch binding properties, especially the key amino acids in the protein for Fch binding.

In the current work, we cloned and purified FtsB and then fully characterized the Fch-binding properties of the protein by using UV-vis, fluorescence and circular dichroism (CD) spectroscopies and stopped-flow kinetics measurements. With the assistances from Accelrys Discovery Studio Client software and site-directed mutagenesis, we firmly showed that Tyr137 and Trp204 are two critical ligands for Fch binding in FtsB and the Fch binding is a two-step process. These findings revealed the detailed Fch-binding features in the Fch-containing proteins in Gram-positive bacteria for the first time.

## Materials and Methods

### Materials

GAS strain MGAS5005 was routinely grown in Todd-Hewitt broth. *Escherichia coli* BL21 (DE3) star strains were purchased from Invitrogen. GST-binding resins were supplied by GE. Centricon-3 and -10 microconcentrators were provided by Millipore. Eight-kilodalton mini dialysis kits were purchased from Merck. Iron-free ferrichrome (Fch), sodium dithionite (Na_2_S_2_O_4_), thrombin and other unspecified chemicals were obtained from Sigma. Trypsin was purchased from Promega.

### Cloning, Expression and Purification of FtsB

The *ftsb* gene without N-terminal signal peptide (residues 1–20) was amplified from the genomic DNA (*S. pyogenes* 5005) by PCR using the forward primer 5′-CGCGGATCCGGTAATCAAGCAACTAATC-3′ and the reverse primer 5′-CGCGTCGACTTAGTTTTCACTTGATAAGATTG-3′ with the *Bam*H I and *Sal* I restriction sites (underlined). The PCR products (873 bp) were digested with *Bam*H I and *Sal* I, and cloned into the GST fusion vector pGEX4T-1. The cloned genes were verified by sequencing and transformed into *E. coli* BL21 (DE3) star cells for subsequent expression.

The transformants were grown to an optical density at 600 nm (OD_600_) of approximately 1.0 at 37°C, followed by induction with 0.5 mM isopropyl β-D-thiogalactopyranoside (IPTG) for 6 h. Bacteria were harvested by centrifugation at 3000* g* for 30 min and lysed by sonication for 20 min. FtsB-GST fusion proteins were isolated by loading whole cell lysate across GST-binding column according to manufacturer’s instruction, verified by SDS-PAGE and Western blotting. The fusion proteins were digested with thrombin (1 µg/U) in PBS buffer overnight at 4°C. Cleaved GST was removed by passages across a GST-binding column. Protein concentrations were determined by the BCA protein concentration assay using BSA as the standard (Bio-Rad). Purified proteins were further confirmed by mass spectrometry using a 4800-plus matrix-assisted laser desorption/ionization time-of-ﬂight mass spectrometer (Applied Biosystems, Foster City, CA, USA). Before analysis, the protein bands were cut off from gels into small pieces and in-gel digested with trypsin overnight at 37°C [12]. Peptide-mass fingerprinting was carried out by searching against the NCBI *S. pyogenes* MAGS5005 database with MS-Fit. The criteria for database matching are: ±25 ppm mass tolerance, at least ten peptides matched, and corresponding molecular weights and pI values.

### Protein 3D Structure Simulation and Binding Site Prediction

Sequence similarity searches of FtsB were initiated by BLAST search tool at NCBI website and analyzed by Clustal-X 2.0. To predict the binding site of Fch in FtsB, the Accelrys Discovery Studio Client 2.5 software was operated. Briefly, the overall structure of FtsB was simulated with the Protein Data Bank (PDB) structure of FhuD (3HXP) in *Bacillus subtilis* (*B.subtilis*) as the search model, and at the same time, all the possible conformations of Fch were modeled. The model rationality by Discovery studio was evaluated by discrete optimized potential energy (DOPE) score, profile-3D score and Ramachandran plot [13]. The DOPE score of a protein structure is regarded as the conformational energy which measures the relative conformation stability with respect to other potential conformations of the same protein. The docking structure of Fch-FtsB was simulated using a flexible docking mode. Then the process of docking was introduced into a simulated environment *in vivo* by adding water, inorganic salt, and atmospheric pressure.

### Site-directed Mutagenesis

E120A, Y137A, W204A and Y287A mutants were constructed by QuikChange mutagenesis kit (Stratagene) with the wild-type *ftsb* plasmid as a template, using primers summarized in Table 1. To further study the collective contribution of Glu120, Tyr137, Trp204 and Tyr287 to the Fch binding of FtsB, a tetra-point mutation E120A/Y137A/W204A/Y287A was produced by mutating these four amino acids to alanine simultaneously. The PCR reactions were performed as follows: denaturation at 95°C for 30 s, followed by 16 cycles with denaturation at 95°C for 30 s, annealing at 55°C for 1 min, and extension at 68°C for 6 min [14]. The mutant plasmids were transformed into *E. coli* XL1-blue for sequencing (Invitrogen). The mutant plasmid with correct sequence was transformed into *E. coli* BL21 (DE3) star competent cells for subsequent expression. Expression and purification of the FtsB mutants were carried out by following the same protocol as for the wild-type protein.

**Table 1:** Primers used for mutant construction.

	Primer
**E120A**	cctcattgttgttggctctacagcagaaaatattaaacaattggcag
**Y137A**	gaaattgcgcctgttatctcgattgaagcccgcaaacgtgatt
**W204A**	cgtttacctttttggtaaagacgcgggacgtggcggaga
**Y287A**	ccatgtgatcaaagtcaatgccaatgtttttgcctttactgatcctctttc

### Fluorescence Spectroscopy

The interaction between FtsB and Fch was investigated in a fluorescence spectroﬂuorimeter (Hitachi F7000), in which the excitation wavelength was 280 nm (slit width of 2.5 nm) and the emission wavelength was between 290 and 500 nm (slit width of 10 nm). Aliquots of Fch (1 mM stock solution) were gradually added to 1 mL FtsB solution (1.5 µM, in 20 mM Tris–HCl, pH 7.2). Fluorescence spectra were collected after each titration until the spectra were stable. The data were analyzed with Hill plot in Origin 7.5 to acquire the binding capability and dissociation constant (*K*_d_). pH dependent Fch binding with FtsB was also examined in the same buffer with pH 3.2–9.2.

### Stopped-flow ﬂuorescence Spectroscopy

Binding kinetics experiments were carried out using a stopped-ﬂow ﬂuorescence spectrometer (Applied Photophysics, SX200) at 25°C. The excitation wavelength was 280 nm and the ﬂuorescence emission was measured using a high-pass filter with a 320 nm cutoff. Binding reaction was initiated by mixing 10 µM FtsB in 20 mM Tris-HCl (pH 7.2) with Fch in a 1∶1 ratio. Kinetics experiments were performed 5 times, and data were analyzed with a second-order or first-order exponential function offered by Origin 7.5 software.

### Circular Dichroism (CD) Spectroscopy

For CD experiments, FtsBs were dialyzed into 20 mM Tris–HCl buffer (pH 7.2) with the final concentration of 3 µM. CD spectra were recorded for FtsB in the absence or presence of 1 molar equivalent of Fch at room temperature using an Applied Photophysics Chriscan spectrometry with a 0.1 cm pathlength quartz cuvette. For secondary structure characterization, CD spectra were recorded from 190 to 260 nm in stepwise of 1 nm at a scan rate of 120 nm/min. Three scans were averaged for each spectrum, and the spectrum of the buffer as the reference was subtracted. Estimations of the protein secondary structures were made using the CDPro software package [15].

### Trypsin Digestion

Fch-FtsB complexes were prepared by incubation of WT and mutant apo-FtsB proteins with 2 molar equivalent of Fch in 20 mM Tris-HCl (pH 7.2) at room temperature for 2 h, followed by the subsequent dialysis of the excess Fch. Trypsin was added to the Apo- and Fch-FtsB solutions at 37°C with a 1∶25 ratio. Fractions (∼10 µg) were removed at 0 min, 5 min, 1, 5, 10 and 20 h respectively and phenylmethanesulfonyl fluoride (PMSF: 0.5 µL, 100 mM) was added to stop the reactions. The mixtures were kept at −80°C for the subsequent SDS-PAGE analysis.

### Native Gel Assay of Ferrichrome Binding

The apo-form FtsBs and Fch-FtsB complexes were subjected to migration assay in Blue-Native gel according to a previously described protocol [16]. Due to the basic p*I* of the proteins, the cathode buffer containing 100 mM histidine (pH 8.0) and the anode buffer containing 100 mM Tris-HCl (pH 8.8) were used. The gel was run for 4 h on ice, and at a half of the time, the cathode buffer without Blue G was applied. The gels were scanned with an Image Scanner (GE healthcare).

## Results

### Structure Modeling of FtsB and Ferrichrome Binding Site Prediction

**Figure 1:**
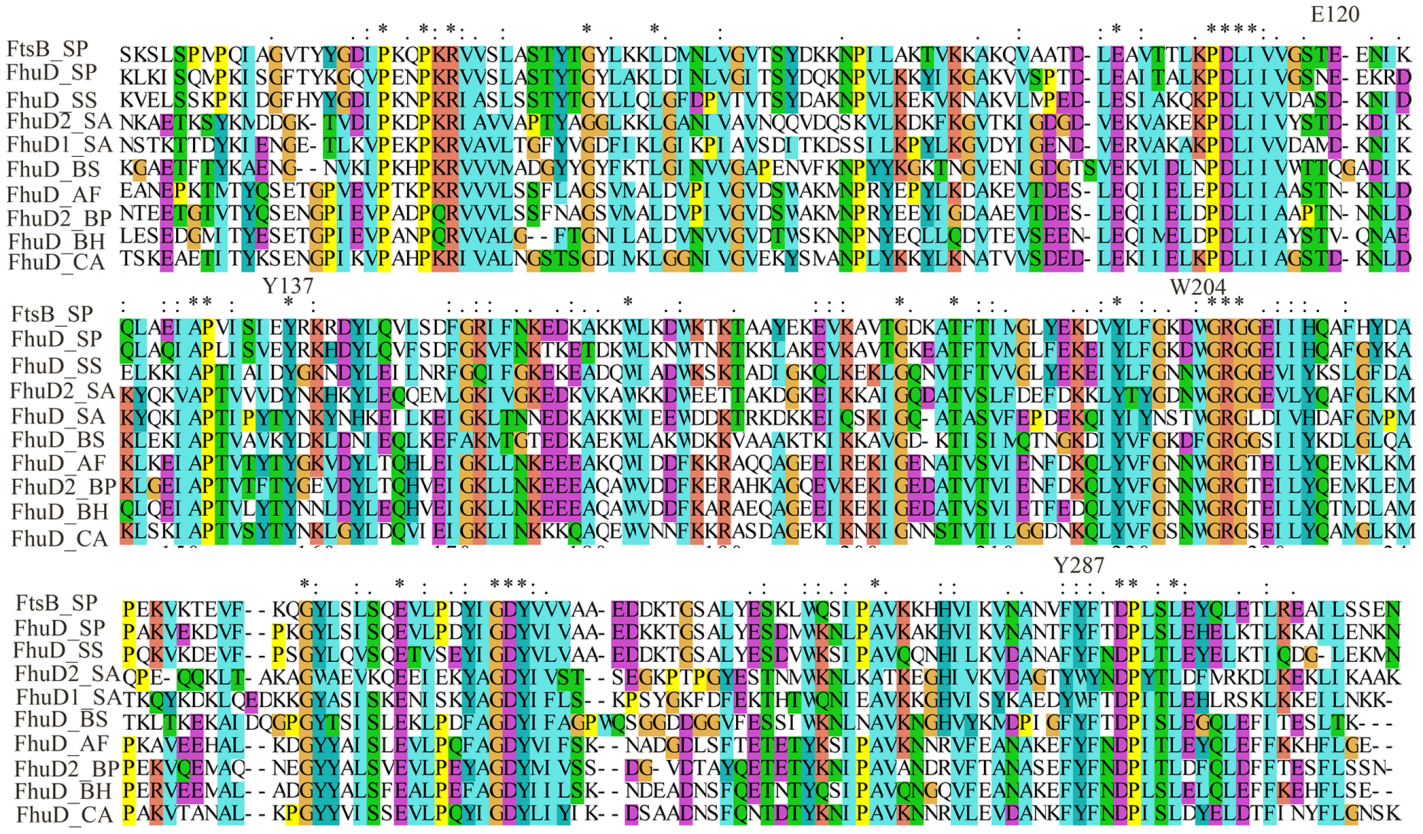
Sequence alignment of FtsB homologs using Clustal-X. Sequences of FhuD1 and FhuD2 from *S. aureus* (SA), FhuD from *B. subtilis* (BS), *B. halodurans* (BH), *C. acetobutylicum* (CA), *S. porcinus* (SP) and *S. sanguinis* (SS) are included. Colours in the alignment are default by the rules as set out in the file colprot.par which comes with the clustal-x application.

To simulate the structure of FtsB and to predict the Fch binding site, the sequence homology analysis was carried out by using NCBI blast tools and Clustal-X. Ten proteins from Gram-positive bacteria are homologous (with at least 33% identity). A multiple sequence alignment showed that 31 amino acids in FtsB are highly conserved in all homologous bacterial proteins (Fig. 1).

FtsB has 37% amino acid sequence identity to FhuD, an Fch-binding lipoprotein from the Gram-positive bacterium *B. subtilis*. The crystal structure of apo-FhuD (3HXP) has been solved and this is the only crystal structure for Fch-binding proteins from Gram-positive bacteria so far. However, the Fch-binding property of the protein remained unknown since this is an Fch-free protein structure.

**Figure 2:**
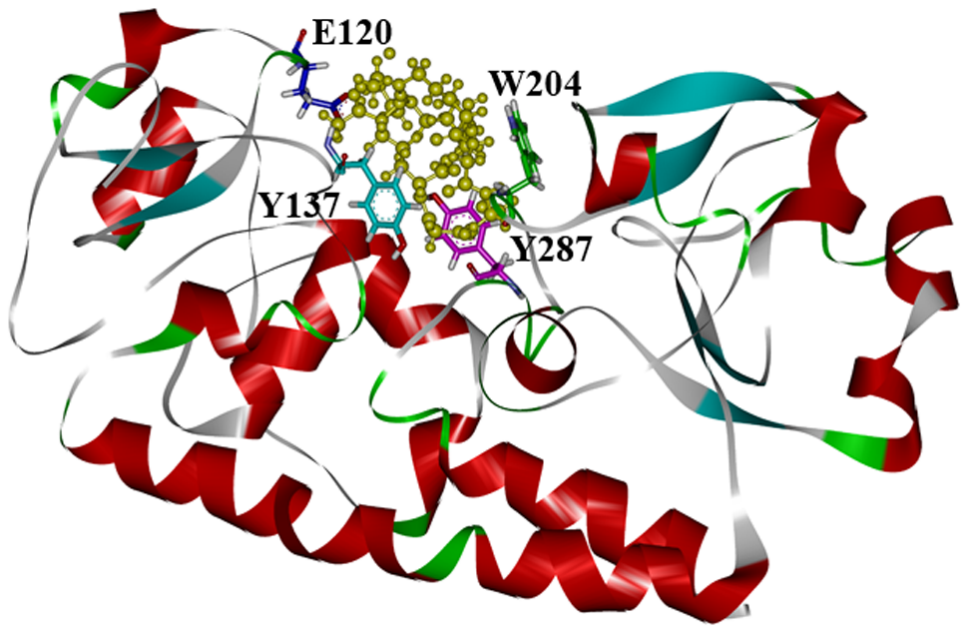
Homology modeling of FtsB. The overall cartoon diagram of FtsB containing ferrichrome (yellow), and the binding sites of Glu120, Tyr137, Trp204, Tyr287 are shown.

On the basis of the crystal structure of apo-FhuD, the FtsB structure was simulated by Discovery studio (Fig. 2). Among the several possible structures, the one with the lowest DOPE score was shown. Ramanchandran Plot and Plot 3D evaluation models showed that the simulated structure of FtsB is confident. The modeled FtsB presents a classic bilobal structural fold of the type III bacterial periplasmic binding protein superfamily, in which the two lobes are linked by a long α-helix of approximately 20 amino acids with a deep cleft for Fch binding [17]. By docking Fch into the FtsB structure, we found that four amino acid residues Glu120, Tyr137, Trp204 and Tyr287, locating on the inner surface of the cleft, interacted with Fch via hydrogen bonds in a hydrophobic environment (Fig. 2). In particular, Tyr137 and Trp204 as two highly conserved residues are respectively located on the N- and C-lobe inside the cleft, while another conserved residue Tyr287 is at the hedge position but Glu120 is from the out-surface (Fig. 2).

### Cloning, Expression and Purification of WT and Mutant FtsB Proteins

**Figure 3:**
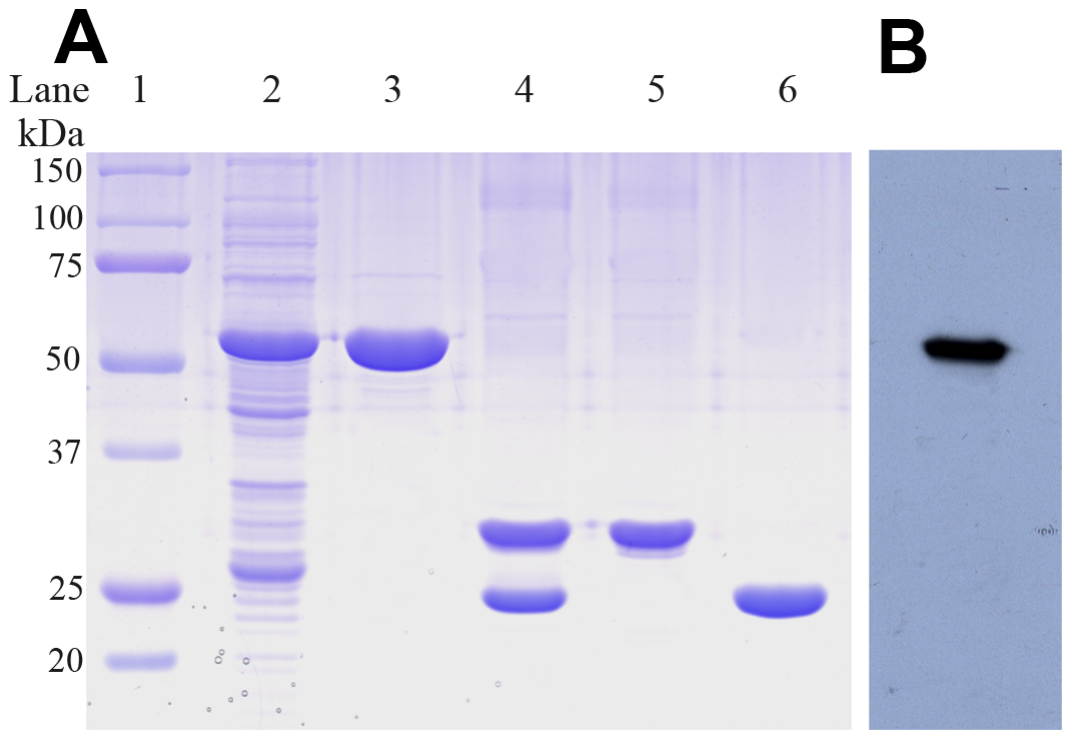
Expression and purification of FtsB. (A) SDS–PAGE analysis of recombinantly expressed FtsB. Lane 1, Molecular marker; lane 2, whole cell lysates; lane 3, fusion protein; lane 4, thrombin cleavage products; lane 5, pure FtsB; lane 6, GST. (B) Western blotting of fusion protein.

The *ftsb* gene fragment (from 61 to 933 bp) was amplified by PCR from genomic *S. pyogenes* 5005 DNA, digested with *Bam*H I and *Sal* I, and then inserted into pGEX4T-1 plasmid. For high-level expression in *E. coli* BL21, the optimal condition is the addition of 0.5 mM IPTG into the culture at an OD_600_ value of ∼1.0, followed by further incubation for 6 h at 37°C. A GST-binding column was used to purify the fusion proteins and then the GST-tags in FtsB-GST were cleaved by thrombin (1 µg/U) overnight at 4°C. The yield of purified FtsB was about 30 mg/L culture. On the 12% SDS-PAGE gel, the purified FtsB migrates as a ∼34 kDa band with over 95% purity (Fig. 3A). Western blotting with anti-GST-tag antibody was performed to verify the recombinant FtsB-GST fusion protein. A single intense band corresponding to the migratory position of the fusion protein was observed on a PVDF membrane (Fig. 3B). FtsB without GST-tag was digested with trypsin and confirmed with MALDI-TOF/TOF mass spectrometry to be correct sequence-wise (Figure S1).

Based on the above structural simulation results, four potential Fch-binding residues Glu120, Tyr137, Trp204 and Tyr287 were individually mutated to be alanine to generate mutant proteins by using site-directed mutagenesis. A tetra-point mutant with the four residues being simultaneously mutated to be alanine was also generated. These mutant proteins E120A, Y137A, W204A, Y287A and Tetra-mutant were expressed and purified with the similar procedure used for the WT FtsB as above and appropriate amounts of pure mutants were obtained (data no shown).

### Ferrichrome Binding Affinities

**Figure 4:**
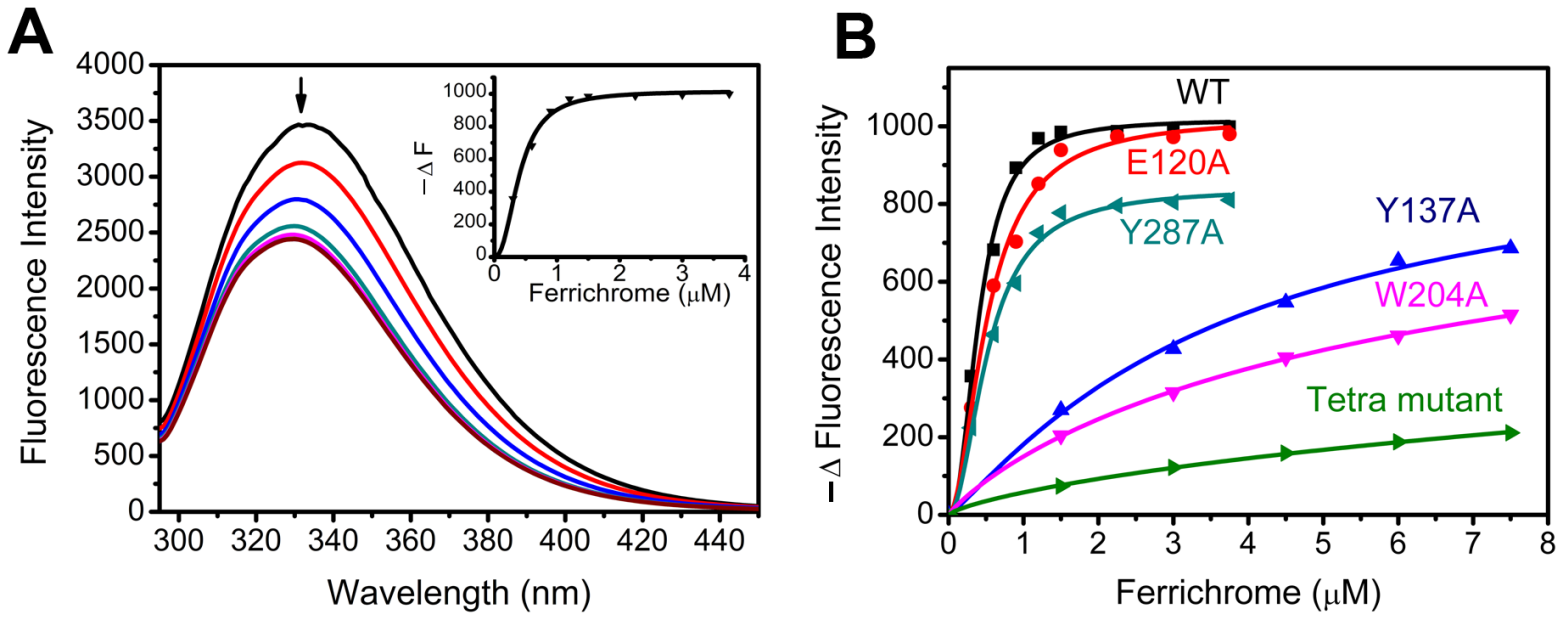
Ferrichrome binding to FtsB. (A) Fluorescence spectroscopy of WT-FtsBs (1.5 µM) in 20 mM Tris-HCl (pH 7.2) upon the addition of aliquot ferrichrome. Apo-WT-FtsB in the presence of 0 (Black), 0.3 (Red), 0.6 (Blue), 0.9 (Dark cyan), 1.2 (Magenata), 1.5 (Dark yellow), 2.25 (Navy), 3.0 (Wine) and 3.75 (Pink) molar equivalents of Fch. (B) Hill plot analysis of the titration curve of ferrichrome with WT and mutant FtsBs.

The interaction between Fch and FtsB was monitored with fluorescence spectroscopy as the addition of Fch to the protein caused a fluorescence quenching at 330 nm (Fig. 4A). Fch binding to FtsB is pH dependent with maximal binding at pH 7.2 (Figure S2). Therefore, pH 7.2 buffer was used in the subsequent experiments. Figure 4A shows that the fluorescence was quenched when aliquots of Fch were titrated into the FtsB solution. Hill plot was used to best fit the function of the fluorescence changes versus the adding amounts of Fch. The fitting result showed that FtsB bound Fch in a 1∶2 ratio and the disassociation constant *K*_d_ was 0.40±0.02 µM. The extra Fch binding in FtsB is probably no-specific, as seen in below the no-specific binding of Fch in the tetra-mutant. Similar method was used to determine the binding affinities of the mutant proteins (Fig. 4B), and the resulting disassociation constants are listed in Table 2. It can be clearly observed from the data shown in Figure 4B and Table 2 that mutants E120A and Y287A had comparable binding affinities with WT FtsB, while mutants Y137A and W204A had much weak Fch binding with approximately 10–20 folds higher *K*_d_ in comparison with WT. Tetra-mutant also showed a little Fch binding, which may be the no-specific interaction with Fch.

**Table 2:** Dissociation Constants for ferrichrome binding with WT and mutant FtsBs.

	WT	E120A	Y137A	W204A	Y287A	Tetra mutant
***K*** **_d_ (µM)**	0.40±0.02	0.52±0.03	3.70±0.10	7.05±0.08	0.52±0.02	43.9±2.5

### Kinetics of Ferrichrome Binding to FtsB

**Figure 5:**
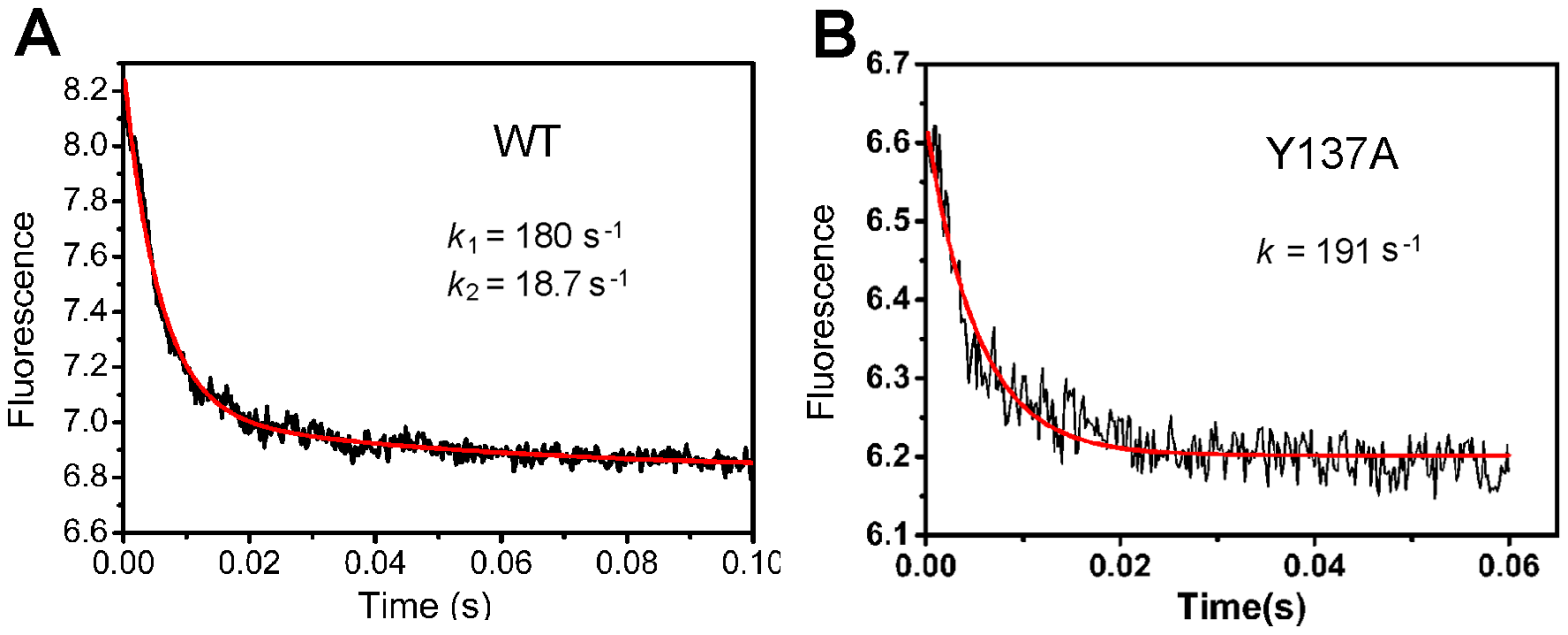
Kinetics of ferrichrome binding to FtsB as measured by stopped-ﬂow ﬂuorometry at 25°C. Fluorescence changes were recorded when WT or mutant FtsB proteins (10 µM) was mixed with ferrichrome in a 1∶1 ratio in 20 mM Tris-HCl (pH 7.2).

Stopped-ﬂow fluorescence spectrometry was used to determine the quick kinetics of Fch binding with FtsB. Figure 5 shows the fluorescence intensity change as a function of time when FtsB reacted with Fch at 25°C. To determine the specific Fch binding rate, FtsB was mixed with Fch in a 1∶1 ratio, but not 1∶2 during the stopped-flow experiment. The process of Fch binding with WT FtsB was composed of a fast phase and a slow phase. The kinetics curve can be well fitted with a second-order exponential function, resulting in a fast rate constant *k_1_* = 180.2±5.4 s^−1^ and a slow rate constant *k_2_* = 18.7±1.3 s^−1^. The binding between Fch and mutant E120A or Y287A produced similar bi-phase kinetics curves with comparable rate constants (data no shown).

Interestingly, the binding kinetics for Fch-Y137A yielded an apparent one-phase curve that can be well fitted with a first-order exponential function, resulting in a rate constant *k* = 191.6±7.6 s^−1^ (Fig. 5B). It can be easily observed that the Fch-Y137A curve showed larger fluctuation due to the smaller overall fluorescence change caused by the mutation of Tyr to Ala in which the intrinsic fluorescence contributed from Tyr137 was eliminated. This situation of data fluctuation was even serious in the case of Fch-W204A so that the kinetics data cannot be properly fitted with any function (Figure S3). This is because that Trp is a bigger contributor than Tyr to intrinsic fluorescence, and the replacement of Trp204 with Ala in W204A led to very small fluorescence change in the Fch-W204A interaction. This phenomenon was also observed in the titration experiments in Figure 4B.

### Effects of Ferrichrome Binding on the Structure of FtsB

**Figure 6:**
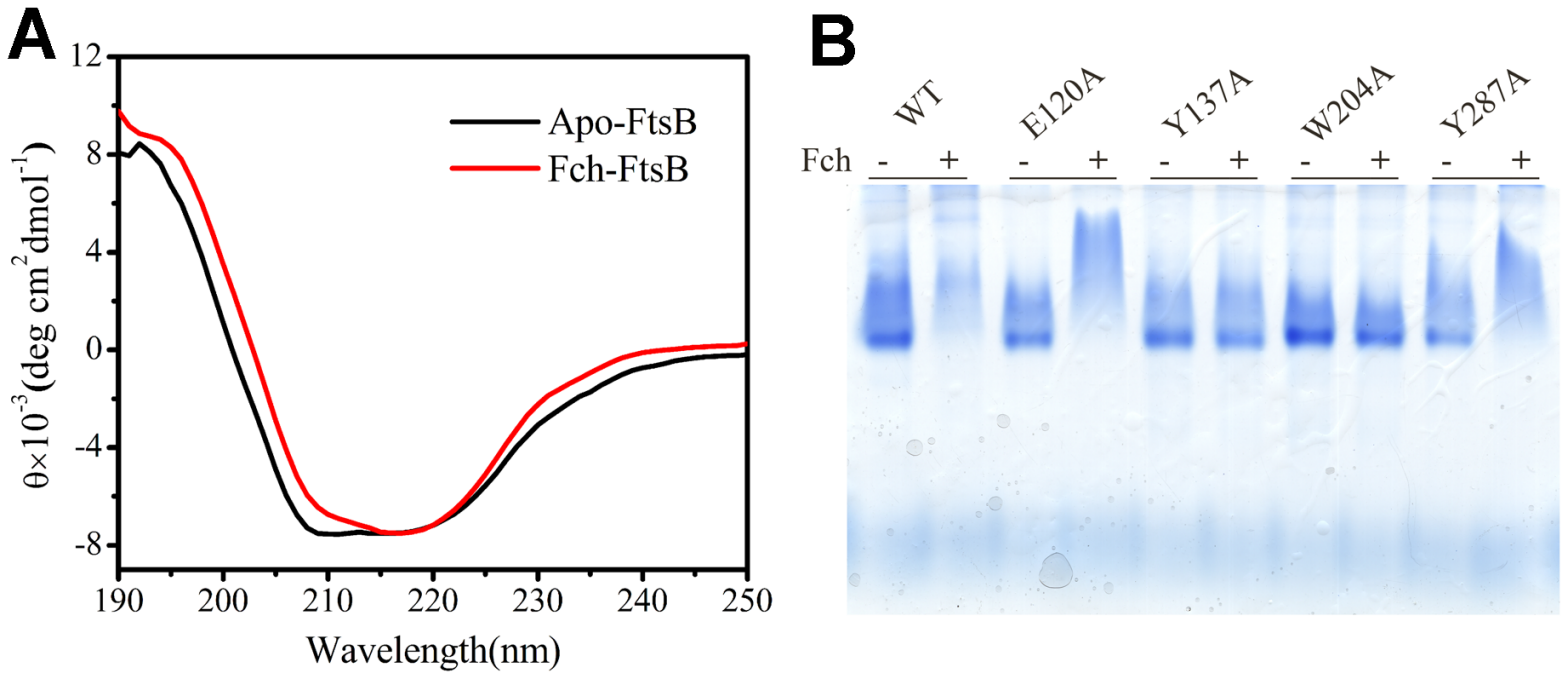
Conformational change of FtsB upon ferrichrome binding. (A) Secondary structure of FtsB (3 µM) as measured by CD spectroscopy in 20 mM Tris-HCl at pH 7.2 with and without ferrichrome. (B) Evaluation of conformational change of FtsB upon ferrichrome binding by Blue-native gel. WT and mutant FtsBs were incubated with ferrichrome for 1 h and analyzed on 12% native gel.

CD spectroscopy was used to monitor the changes of secondary structure upon Fch binding to FtsB. The CD spectrum of apo-FtsB in 20 mM Tris-HCl was recorded and two negative bands at 208 and 222 nm and a positive band at around 192 nm were observed (Fig. 6A). Quantitative analysis using CDPro indicated that α-helix predominates in FtsB (Table 3) and Fch binding induced only minor changes in the secondary structure of FtsB.

**Table 3:** Secondary structural analyses based on circular dichroism spectral measurements.

	α-helix (%)	β-sheet (%)	Turn (%)	Unordered (%)
**Apo-FtsB**	53.9±1.1	19.0±0.3	11.4±0.4	15.2±2.1
**Fch-FtsB**	51.5±2.1	21.8±0.8	10.7±0.7	16.0±0.5
**Apo-E120A**	58.5±1.2	20.4±1.4	10.3±0.5	11.3±0.2
**Fch-E120A**	53.5±1.5	20.0±0.1	14.7±1.2	11.8±1.0
**Apo-Y137A**	70.6±1.8	14.2±1.2	8.3±1.1	7.0±0.5
**Fch-Y137A**	63.1±0.6	15.5±0.6	10.5±0.5	10.7±1.1
**Apo-W204A**	64.2±1.9	16.5±0.3	8.6±0.6	10.5±0.6
**Fch-W204A**	56.5±2.1	20.2±1.6	8.3±2.2	15.0±1.4
**Apo-Y287A**	47.8±0.6	20.1±1.0	12.9±0.3	19.2±0.9
**Fch-Y287A**	45.7±2.3	23.4±0.7	15.0±0.3	15.9±0.8
**Apo-Tetra mutant**	58.6±0. 5	14.0±0.8	10.1±1.2	17.3±0.7
**Fch-Tetra mutant**	51.5±2.1	21.1±2.1	11.5±0.6	15.8±1.0

Triplicate independent measurements were performed.

When CD spectroscopic scans were applied to all the mutant proteins, similar spectral features as in Figure 6A were obtained (data no shown). Table 3 lists the secondary structural compositions as estimated by CDPro for all the CD spectra of the mutant proteins. Overall, mutations at residues Tyr137 and Trp204 rendered larger alterations than mutations at Glu120 and Tyr287 as shown in the CD analyses. Moreover, in comparison with the situation in WT FtsB and mutant Y287A, much more significant conformational changes were observed in the Fch binding with mutants Y137A and W204A.

To further investigate the effects of the conformational change on FtsB, the apo- and Fch-bound forms of FtsBs were analyzed by native gel. As shown in Figure 6B, Fch-bound WT FtsB and its apo-form presented totally different migration patterns on the gel. The severe smearing pattern of apo-FtsB implicates that the apo-protein may have dynamic conformational forms without Fch binding. Mutants E120A and Y287A had similar phenomena with WT FtsB in the gel pattern, suggesting that no significant conformational alteration occurred in the mutations of Glu120 and Tyr287. In contrast, Fch-loaded Y137A and W204A mutants migrated on the native gel in manners apparently distinct from WT Fch-FtsB, resembling their apo-forms in patterns (Fig. 6B). These observations indicate that mutations at Tyr137 and Trp204 proceeded a remarkable impact on the conformation and the Fch binding of FtsB.

### Proteolytic Stability of FtsB

**Figure 7:**
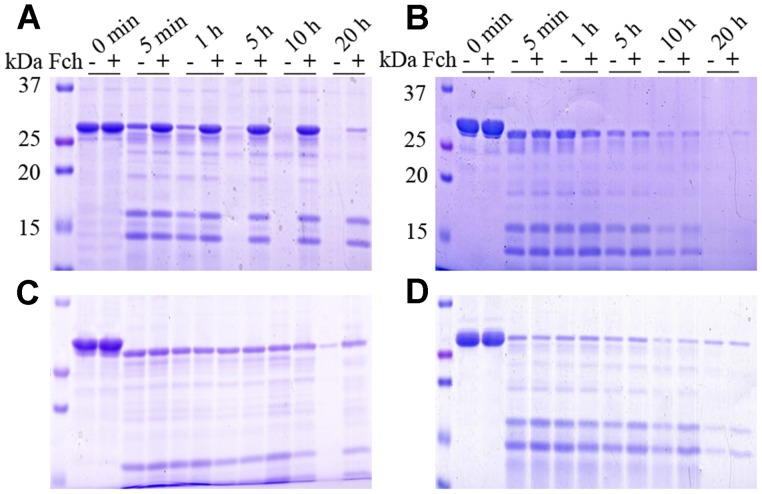
Tryptic digestion of apo- and ferrichrome-bound FtsBs. FtsBs were digested with trypsin (1∶25) at 37°C in the absence (–) and presence (+) of two molar equivalents of ferrichrome at indicated time and analyzed on 15% SDS-PAGE. A–D represent WT, Y137A, W204A, and Tetra mutant, respectively.

Trypsin was used as a proteinase to study the proteolytic stability of FtsB. As shown in Figure 7A, apo-FtsB can be gradually cleaved by trypsin from 34 kDa into two major fragments, around 17 and 13 kDa, respectively, and be completely digested after 5 h. Fch-bound FtsB showed much more resistance to the trypsin proteolysis, with most of the molecule at 34 kDa still standing at 10 h (Fig. 7A), suggesting that Fch binding renders a more stable structure against trypsin digestion. The stabilities of FtsB mutants in the Fch-bound forms were compared with that of WT protein using the same method (Figs. 7B–D). Mutants E120A and Y287A also showed resistances to trypsin digestion with cleavage patterns similar to WT FtsB (data not shown). However, mutants Y137A, W204A and Tetra-mutant were much labile in the proteolysis; most proteins were digested in the initial 5 h, similar to their apo-forms (Figs. 7B–D).

## Discussion

Fe^3+^-siderophores including Fch can be transported across the cytoplasmic membrane in both Gram-positive and -negative bacteria by ABC transporters [18]. FhuABCD transporter is a classical Fch uptake system that presents in the majority of both Gram-positive and -negative bacteria. So far, FhuD in the FhuABCD from Gram-negative *E. coli* is the only Fch-binding protein with solved crystal structure, in which two key residues Arg84 and Tyr106 form hydrogen bonds with gallichrome (Fch analogue) in a hydrophobic cleft [19]. However, these two key residues are not conservative in FhuDs from Gram-positive bacteria (Fig. 1). A recent crystal structure of apo-FhuD (3HXP) from Gram-positive *B. subtilis* confirmed the absence of the residues Arg84 and Tyr106 [17], [20]. This difference in conservative key residues implicates that FhuD in Gram-positive bacteria may have a distinct Fch binding mechanism from that in Gram-negative bacteria.

Using the crystal structure of FhuD as a model, the structure of FtsB from *S. pyogenes* was simulated in this study, which features two independent lobes of mixed α/β structure separated by a deep cleft for Fch binding (Fig. 2). The secondary structure of FtsB as calculated by the CD spectra revealed that α-helix is the predominant component in the protein, and a long α-helix linker forming a hedge restricts the movement of the two lobes, resulting in a small conformational change upon Fch binding (Fig. 6A) [17]. Even though, Fch binding with the small conformational change significantly stabilized the structure of FtsB, as demonstrated by trypsin digestion (Fig. 7A), chemical and thermal denaturation experiments (data no shown).

According to the structural modeling, residues Glu120, Tyr137, Trp204 and Tyr287 are four potential ligands for Fch binding in FtsB. Previous biochemical study revealed that Tyr191, Trp197 and Glu202 played important roles in Fe^3+^-hydroxamate binding of FhuD in *S. aureus* and group B *streptococcus* [21], [22]. In the sequence homology, Trp197 of FhuD corresponds to Trp204 of FtsB in *S. pyogenes*. Accordingly, residue Trp204 is probably a conservative Fch binding ligand in FtsB. This speculation was evidently confirmed by the fact that elimination of the ligation ability from Trp204 in the mutant W204A significantly decreased the Fch-binding affinity of the protein (Fig. 4B).

In addition, we first found in this study that residue Tyr137 is another essential ligand for Fch binding in FtsB; without Tyr137, mutant Y137A showed much diminished Fch-binding ability (Fig. 4B). Although Tyr137 is a highly conservative amino acid in Gram-positive bacteria FhuDs, there is not any report about its function. The importance of Tyr137 and Trp204 in the protein was also presented in the stability against proteolysis; lacking any of these two ligands led to labile structures easy to be digested by trypsin (Fig. 7). On the other hand, residues Glu120 and Tyr287 may not be the key ligands for Fch binding, as mutants E120A and W287A demonstrated similar properties to the WT FtsB in Fch-binding affinity, native-gel migration and proteolysis resistance (Figs. 4, 5, 6, 7).

Another important finding in this work is the observation that Fch binding in FtsB is a two-step interaction, probably corresponding to the Fch bonding with the two essential ligands in stepwise. This can be easily deduced from our kinetics experimental data in which WT FtsB and mutants E120A and W287A carried out bi-phase reactions whereas mutant Y137A presented in one-step process (Fig. 5). More interestingly, curve fitting results showed that Y137A had an Fch binding constant *k* = 191 s^−1^, resembling the fast rate of *k*_1_ = 180 s^−1^ in the Fch binding of WT FtsB (Fig. 5). These observations suggest that Fch binding in the protein may proceed with residue Tyr137 first and then with residue Trp204.

As shown in Figures 1 and 2, Tyr137 and Trp204 are two highly conserved residues located on the inner surfaces of the binding cleft, and are respectively from N- and C-lobe composing the cleft. It is therefore possible that Fch initially binds to the N-lobe using Tyr137 as an anchor and then binds Trp204 from the C-lobe, closing the cleft of the protein. The conformational change resulting from the cleft closing is although small but critical to the protein. Without one of the essential ligands Tyr137 and Trp204, the cleft may not be fully closed in the Fch binding, leading to somehow “sampling” conformations, and thus mutants Y137A and W204A migrated as apo proteins in native gels (Fig. 6).

In conclusion, the lipoprotein FtsB was cloned from *S. pyogenes* and the Fch binding properties of the protein were characterized in detail. We confirmed that residue Trp204 is a key ligand for Fch binding and identified that residue Tyr137 is another essential ligand. We further demonstrated that Fch binding in FtsB is a two-step process corresponding to the interactions to Tyr137 in the N-lobe and Trp204 in the C-lobe in stepwise. The inconsistence in sequence identities among Fch-binding proteins from Gram-positive and –negative bacteria renders significantly different binding environments in the proteins, probably leading to distinct iron-uptake mechanism. As the first detailed biochemical characterization of the Fch-binding proteins from Gram-positive bacteria, the current work provides valuable information for understanding the differentiations in terms of the structure-function relationships in Fch-binding proteins from bacteria.

## Supporting Information

Figure S1 Mass spectrum of purified FtsB (upper panel) and database searching report (lower panel).
(TIF)

Figure S2 pH dependence of Fch binding with FtsB.
(TIF)

Figure S3 Kinetics curve of Fch binding with W204 mutant.
(TIF)
