# Supplementary figures and images for: Lipoprotein FtsB in Streptococcus pyogenes Binds Ferrichrome in Two Steps with Residues Tyr137 and Trp204 as Critical Ligands
Source: PLoS One. 2013 Jun 20;8(6):e65682. doi: 10.1371/journal.pone.0065682 (PMC3688767; doi:10.1371/journal.pone.0065682)

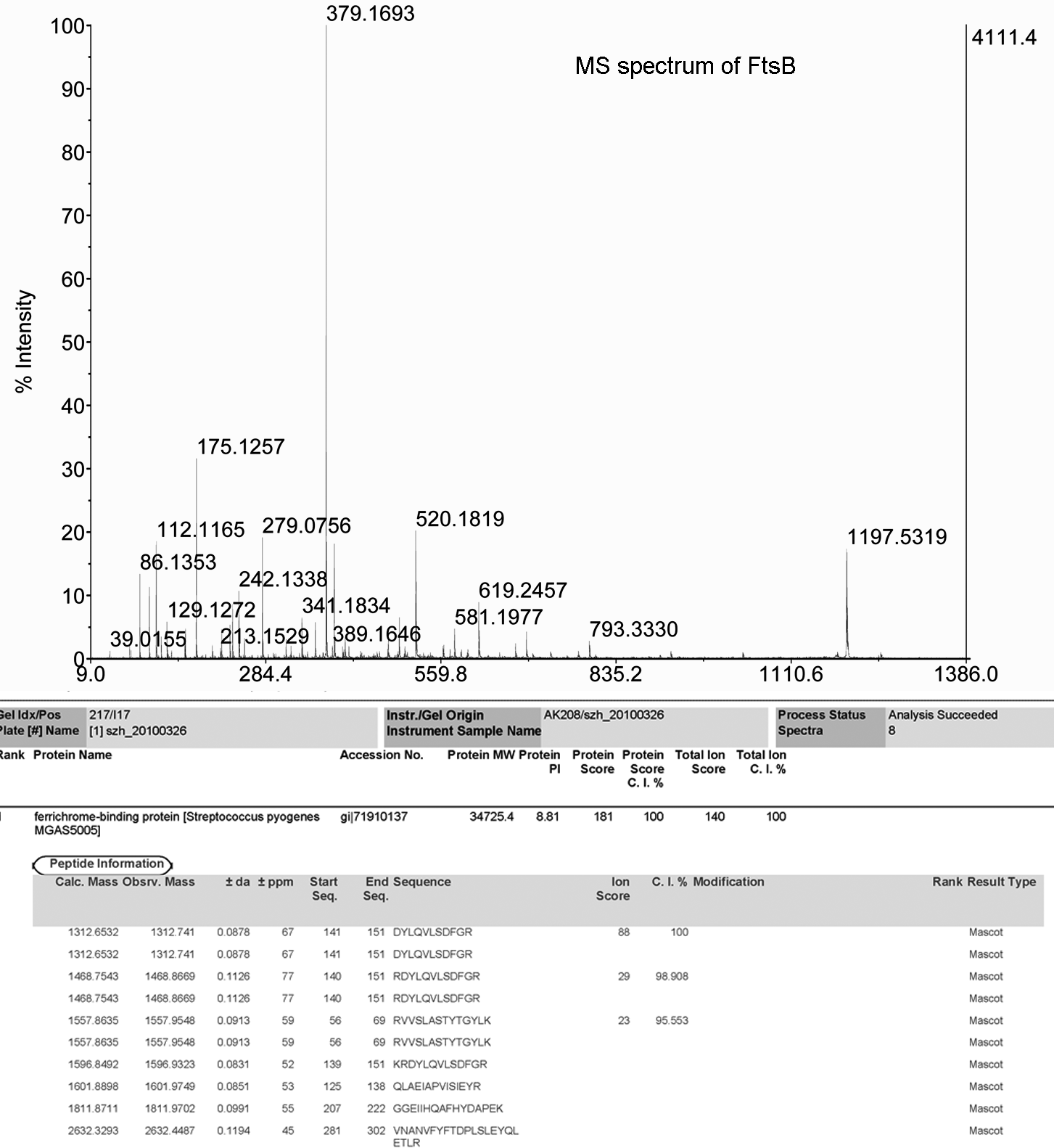

Supplement: Figure S1 — Mass spectrum of purified FtsB (upper panel) and database searching report (lower panel). (TIF) [file pone.0065682.s001.tif]
